# Randomised trial of intravenous thiamine and/or magnesium sulphate administration on erythrocyte transketolase activity, lactate concentrations and alcohol withdrawal scores

**DOI:** 10.1038/s41598-022-10970-x

**Published:** 2022-04-28

**Authors:** Donogh Maguire, Alana Burns, Dinesh Talwar, Anthony Catchpole, Fiona Stefanowicz, David P. Ross, Peter Galloway, Alastair Ireland, Gordon Robson, Michael Adamson, Lesley Orr, Joanna-Lee Kerr, Xenofon Roussis, Eoghan Colgan, Ewan Forrest, David Young, Donald C. McMillan

**Affiliations:** 1 Emergency Medicine Department, Glasgow Royal Infirmary, 84 Castle Street, Glasgow, G4 0SF UK; 2 Academic Unit of Surgery, School of Medicine, University of Glasgow, New Lister Building, Royal Infirmary, Glasgow, G31 2ER UK; 3 Department of Clinical Biochemistry, Queen Elizabeth University Hospital, Govan, G51 4TF UK; 4 The Scottish Trace Element and Micronutrient Diagnostic Reference Laboratory, Department of Biochemistry, Royal Infirmary, Glasgow, G31 2ER UK; 5 Department of Gastroenterology and Hepatology, Glasgow Royal Infirmary, Glasgow, G4 0SF UK; 6 Department of Mathematics and Statistics, University of Strathclyde, Richmond Street, Glasgow, G1 1XH UK

**Keywords:** Medical research, Biochemistry, Enzyme mechanisms

## Abstract

Alcohol withdrawal syndrome (AWS) occurs in 2% of patients admitted to U.K. hospitals. Routine treatment includes thiamine and benzodiazepines. Laboratory studies indicate that thiamine requires magnesium for optimal activity, however this has not translated into clinical practice. Patients experiencing AWS were randomized to three groups: (group 1) thiamine, (group 2) thiamine plus MgSO_4_ or (group 3) MgSO_4_. Pre- and 2-h post-treatment blood samples were taken. AWS severity was recorded using the Glasgow Modified Alcohol Withdrawal Score (GMAWS). The primary outcome measure was 15% change in erythrocyte transketolase activity (ETKA) in group 3. Secondary outcome measures were change in plasma lactate concentrations and time to GMAWS = 0. 127 patients were recruited, 115 patients were included in the intention-to-treat analysis. Pre-treatment, the majority of patients had normal or high erythrocyte thiamine diphosphate (TDP) concentrations (≥ 275–675/> 675 ng/gHb respectively) (99%), low serum magnesium concentrations (< 0.75 mmol/L) (59%), and high plasma lactate concentrations (> 2 mmol/L) (67%). Basal ETKA did not change significantly in groups 1, 2 or 3. Magnesium deficient patients (< 0.75 mmol/L) demonstrated less correlation between pre-treatment basal ETKA and TDP concentrations than normomagnesemic patients (R^2^ = 0.053 and R^2^ = 0.236). Median plasma lactate concentrations normalized (≤ 2.0 mmol/L) across all three groups (*p* < 0.001 for all groups), but not among magnesium deficient patients in group 1 (n = 22). The median time to achieve GMAWS = 0 for groups 1, 2 and 3 was 10, 5.5 and 6 h respectively (*p* < 0.001). No significant difference was found between groups for the primary endpoint of change in ETKA. Co-administration of thiamine and magnesium resulted in more consistent normalization of plasma lactate concentrations and reduced duration to achieve initial resolution of AWS symptoms.

ClinicalTrials.gov: NCT03466528.

## Introduction

In 2018, the WHO reported that harmful alcohol use accounted for 5% of deaths worldwide and 10% of deaths per year in the European region^1^. Currently, one in five patients admitted into the UK hospital system satisfy criteria for diagnosis of alcohol use disorder (AUD)^2^. Previous reports estimate that 10% of patients who satisfy the criteria for diagnosis of AUD will develop alcohol withdrawal syndrome (AWS) during their hospital admission^3^.

**Figure 1:**
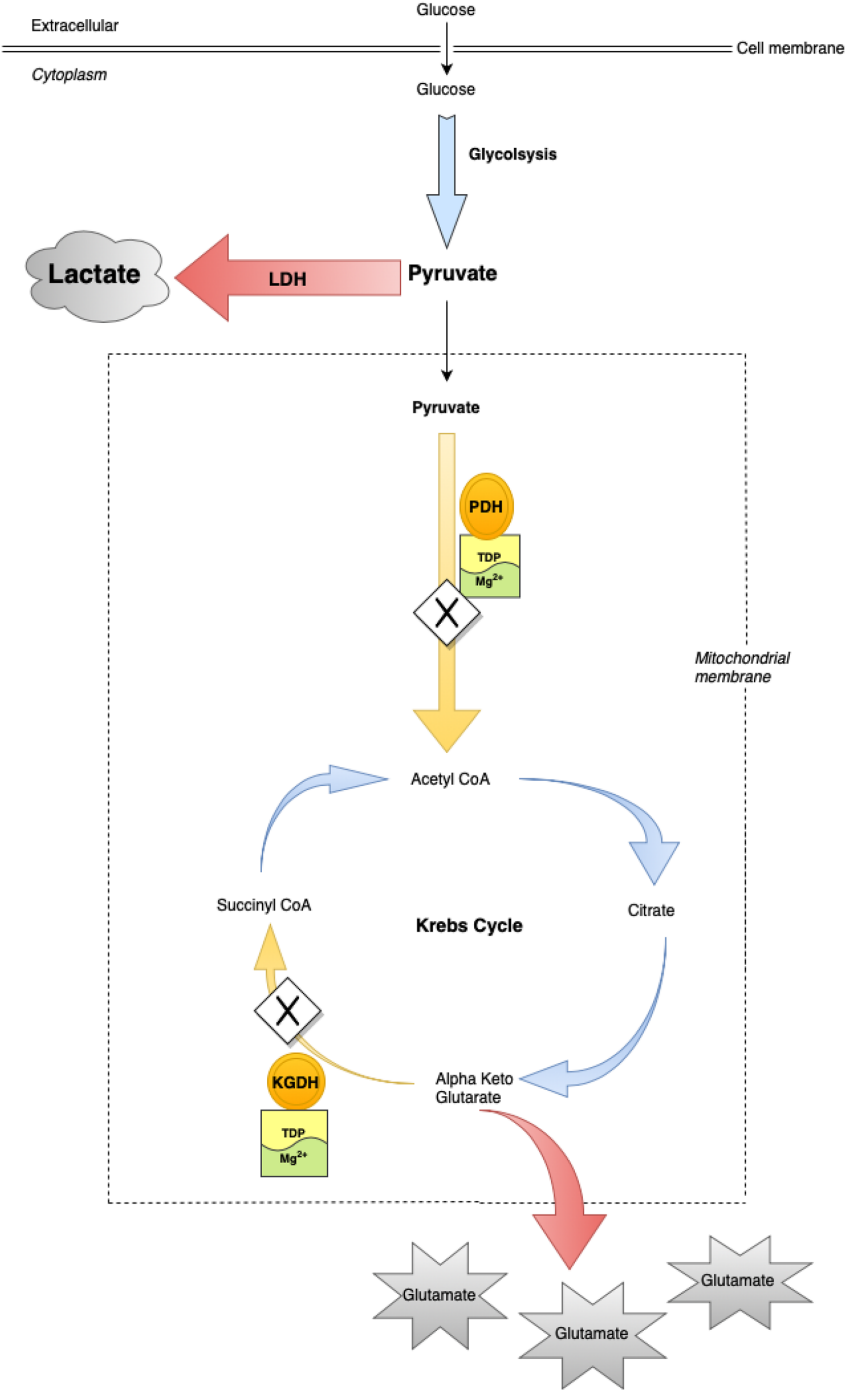
Pseudo-hypoxic ‘Dirty burn’ metabolism resulting in increased lactate production during AWS.

It has recently been established that serum magnesium concentrations may be low in patients with AWS and independently associated with survival at 1-year^4,5^. In contrast, thiamine concentrations were in the normal range and not independently associated with poorer survival at 1-year^5^. This study also reported that > 60% patients attending the Emergency Department with AWS had plasma lactate concentrations > 2.0 mmol/L^5^. Thiamine and magnesium are recognised to be cofactors in a number of enzymes such that they may influence the formation of lactate^5^. Thiamine and magnesium act as essential co-factors for a family of three mitochondrial dehydrogenases [pyruvate dehydrogenase (PDH), alpha-ketoglutarate dehydrogenase (KGDH) and branched chain keto-acid dehydrogenase (BCKDH)] that orchestrate efficient mitochondrial aerobic ATP production^6^. Therefore, in the context of normal partial pressure tissue oxygen concentrations and in the absence of an inflammatory response or intense skeletal muscle activity (e.g. alcohol withdrawal seizure), thiamine and/or magnesium deficiency mediated increase in metabolism of glucose to lactate may represent pseudo-hypoxic disruption of normal glucose metabolism (‘Dirty burn metabolism’) (Fig. 1)^5,7,8^.

Thiamine and magnesium also serve as co-factors for the cytoplasmic enzyme, transketolase (TK), which catalyses the reversible arm of the Pentose Phosphate Pathway (PPP). TK has a central role in the generation of reducing equivalents for the maintenance of the cellular redox state^9^.

Biochemical evidence of thiamine deficiency may manifest directly with reduced mass of thiamine in erythrocytes, or indirectly with reduced activity of the functional enzymatic marker of thiamine status, erythrocyte transketolase activity (ETKA)^10^. Furthermore, increased plasma lactate concentrations may occur with thiamine and/or magnesium deficiency^11–15^.

Interestingly, magnesium (Mg^2+^) is required for optimal absorption of thiamine from the gut, activation of free thiamine to active phosphorylated form (thiamine diphosphate (TDP)) and optimum activity of TDP dependent enzymes^16^. This biochemical interdependence has been understood for decades, however only one small study has examined the in vivo effect of concomitant administration of thiamine with magnesium^17^. A Cochrane review concluded that there was insufficient evidence as to the duration of treatment and dosages of thiamine required for the treatment/prevention of Wernicke’s encephalopathy (WE), while acknowledging that thiamine utilization in patients experiencing alcohol withdrawal syndrome is probably influenced by Mg^2+^ status^18^. Moreover, another Cochrane review concluded that there was insufficient evidence to currently support the routine administration of magnesium to patients suffering acute alcohol withdrawal syndrome^19^.

Based on a previous pilot study that showed significant change in thiamine dependent enzyme activity (ETKA) when patients (n = 36) with AWS were treated with combined thiamine and magnesium versus thiamine alone^17^, the primary endpoint of change in ETKA and secondary endpoint of change in plasma lactate concentrations were chosen for the present trial. The initial pilot study had significant limitations however as baseline whole blood thiamine, serum magnesium, or plasma glucose and lactate concentrations were not measured.

### Patients and methods

To better understand the clinical practicalities and biochemical effects of concurrent thiamine and magnesium supplementation on ETKA, plasma lactate concentrations and rate of resolution of alcohol withdrawal syndrome, an investigation of a cohort of AUD patients presenting to the Emergency Department (ED) with symptoms of alcohol withdrawal syndrome was undertaken. All participants were recruited to the study following presentation to the Emergency Department with symptoms or signs of alcohol withdrawal syndrome. Patients with a background history of heart failure or renal impairment were excluded.

The trial protocol was approved by the West of Scotland Research Ethics Committee (16/WS/0162) and registered with clinicaltrials.gov (NCT03466528) (15/03/2018). All methods and procedures were carried out in accordance with relevant guidelines and regulations^20^. Patients were recruited in the ED at Glasgow Royal Infirmary by convenient enrollment between 1/1/2017 and 1/6/2018 and followed up for one year thereafter. The trial ended when recruitment met last patient/last visit criteria.

Informed consent was received prior to administration of questionnaire and venesection. Randomization was performed by the trial statistician using the sealed envelope programme: https://www.sealedenvelope.com/simple-randomiser/v1/lists

Opaque envelopes detailing the subject number and treatment allocation were prepared. Patients were sequentially assigned a subject number once eligibility was confirmed and the corresponding treatment assignment envelope opened. The opened enveloped was retained and attached to the patient’s case report form (CRF). Patients were blinded to randomization allocation.

Patients who were unable to give informed consent or < 18 years of age were excluded. Patients with a history of chronic renal or hepatic failure, hepatic encephalopathy; known hypersensitivity or previous allergy to any of the active substances in either trial medication; or severe concurrent medical condition that would prevent participation in trial procedures (e.g. myasthenia gravis, clinically significant cardiac disease, or cardiac failure with severe pulmonary oedema) were also excluded.

Male or non-pregnant or breastfeeding females ≥ 18 years of age who were capable of giving informed consent and confirmed to be experiencing alcohol withdrawal syndrome (AWS) were approached for trial participation.

AWS was determined by the Glasgow Modified Alcohol Withdrawal Scale (GMAWS) and standardized benzodiazepine (BDZ) treatment was administered as per the GMAWS protocol^21,22^.

Thiamine in its commercial form, Pabrinex® (U.K.), was administered intravenously to patients with or without magnesium sulphate (MgSO_4_) 2 g supplementation over 30 min duration according to randomization status. Pabrinex® is a multivitamin preparation that is equivalent to the “banana bag” administered in U.S. Emergency Departments.

Patients were randomized to receive one of three treatments: (group 1) standard treatment (thiamine 250 mg in 100 mL of saline over thirty minutes) or (group 2) standard treatment plus MgSO_4_ 2 g (infused in the same bag of crystalloid over 30 min duration) or (group 3) MgSO_4_ 2 g (infused in 100 mL of saline over thirty minutes). Blood samples were taken for analysis prior to, and 2 h post administration of thiamine+/MgSO_4_. Patients who received MgSO_4_ alone (Group 3) received standard treatment (thiamine 250 mg in 100 mL of saline over thirty minutes) immediately after the 2-h trial samples were drawn. Intravenous fluid administration was standardized for all patients (500 mL normal saline over 2 h).

Standard serum separating tubes were used for routine serum biochemistry, EDTA tubes were used for both routine and erythrocyte thiamine diphosphate sample collection and trial samples were collected in non-gel lithium heparin tubes for measurement of ETKA and erythrocyte magnesium concentrations. All samples were conveyed to the laboratory within 1 h of collection. Routine biochemistry and hematology samples were analyzed contemporaneously. Whole blood EDTA samples for thiamine diphosphate measurement were frozen at − 70 °C and analyzed within ten days of being drawn. Trial samples for measurement of ETKA and erythrocyte magnesium were centrifuged (500G for 10 min), and plasma was carefully removed. Erythrocytes were washed three times prior to storage. Both the separated plasma and packed erythrocytes were stored at − 70 °C until analysis. All samples from any given patient were assayed in a single batch for each of the analytes to minimize inter-batch analytic variation^23^.

Routine ETKA measurement (in vitro TDP enhanced ETKA measurement) is a functional marker of thiamine status that measures the percentage change in ETKA when TDP is added to the assay, relative to basal ETKA^23^. The percentage increase in transketolase activity following addition of TDP represents the patient’s thiamine status (> 15% and > 25% indicating moderate and severe thiamine deficiency respectively). For the present trial, in addition to routine ETKA measurement (in vitro TDP enhanced ETKA measurement), pre- and post-treatment basal ETKA has also been reported to examine the relative change of ETKA in response to the randomized treatment administered.

### Analytical methods

Measurement of thiamine diphosphate in erythrocyte involved HPLC isocratic separation with post-column derivatization using sodium hydroxide and potassium ferricyanide and fluorescent detection. Results were expressed as nanogram of thiamine diphosphate per gram of haemoglobin (ng/gHb)^10,23^.

Erythrocyte transketolase (ETKA) was measured by adapting the method developed by Bayoumi and Rosalki^24^. Briefly, ETKA was determined by a two-step coupled enzymatic reaction both with and without the in vitro addition of TDP (ETKA-TDP and basal ETKA respectively). The reduction of NADP^+^ to NADPH was measured spectrophotometrically at 340 nm over 30 min to calculate ETKA in U/gHb. Haemoglobin for both thiamine diphosphate and ETKA methods were determined by the cyano-methaemoglobin assay^25^:

$${\text{Hb }}\left( {{\text{g}}/{\text{L}}} \right){ } = { }\frac{{{\text{Iron }}\left( {{\text{mol}}/{\text{L}}} \right)}}{4} \times 64{,}456$$

Erythrocyte magnesium was measured using inductively coupled plasma mass spectrometry on the Agilent 7900 ORS-ICP-MS (Agilent Technologies, Santa Clara, California, United States). Erythrocyte magnesium was reported as mmol per gram of haemoglobin (mmol/gHb) to mitigate pipetting errors that are commonly associated with pipetting packed erythrocytes due to their viscosity^26^. Haemoglobin concentration in the sample was derived using the ^57^Fe concentration and the above equation^23,26^.

Serum C-reactive protein (CRP), albumin and magnesium were measured in accordance with the manufacturer’s instructions, by routine laboratory procedures, on an automated analyzer (Architect; Abbott Diagnostics, Abbott Park, Chicago, IL). For C-reactive protein, the limit of detection was 1 mg/L. The inter-assay CV was < 5% over the sample concentration range for the analytes measured^23^.

### Sample size

Based on pilot study data that showed significant change in thiamine dependent enzyme activity (ETKA) when patients (n = 36) with AWS were treated with combined thiamine and magnesium versus thiamine alone^17^, the primary endpoint of 15% change in ETKA in the magnesium only arm was chosen for the present trial. A three-group trial was designed to detect a minimum biochemically significant difference of 0.16 U/gHb change from baseline between the groups, with 80% power and a 5% significance level. This required 37 participants to be randomised to each arm of the trial. A total sample size of 120 was chosen to account for any dropouts or missing data.

### Statistical analysis

Data are presented as medians and inter-quartile ranges (IQR). Categorical data were analysed using chi-squared and McNemar’s tests; continuous data were analysed using Mann–Whitney or Kruskal–Wallis tests. Wilcoxon’s signed ranks test was used to analyse the pre- and post-treatment difference between paired samples within randomization groups. Repeated measures ANOVA was used to analyse the pre- and post-treatment difference between randomization groups and post-hoc comparisons were done using Tukey and Dunnett’s tests. Correlations were calculated using Spearman’s correlation (r_s_). All analyses were done using SPSS software (version 27, SPSS Inc, Chicago, IL) at a 5% significance level. Owing to the number of statistical comparisons carried out (~ 45), a *p* value ≤ 0.01 was considered statistically significant.

## Results

All patients (n = 127) recruited to the trial fulfilled the criteria for inclusion with age, sex, BMI and objective evidence of alcohol withdrawal syndrome or scored positively on FAST questionnaire indicating Alcohol Use Disorder. Twelve patients were recruited more than once and therefore excluded from the analysis after the first attendance resulting in a total of 115 patients included in the baseline analysis; 3 patients opted out of the trial prior to administration of the trial drug and self-discharged from the Emergency Department. All 115 patients are included in the intention to treat analysis^27^.

The clinicopathological characteristics are shown in Table 1. The majority of patients were < 50 years old (64%), male (77%), not underweight (81%), had a severe alcohol withdrawal score (GMAWS max score ≥ 4) (65%) and the total ‘diazepam-equivalent’ dose of benzodiazepine (BDZ) administered was more than 120 mg (52%). The majority of patients reported recent weight loss (67%) and difficulty walking (83%). The median ‘time since last drink’ was 20 h (IQR 13–39 h). No significant differences in baseline characteristics were found between the three-randomization groups (Tables 1, 2).

**Table 1:** Baseline characteristics of patients recruited to the randomised controlled trial (n = 115).

	Group 1 Thiamine (n = 38)	Group 2 Thiamine and Magnesium (n = 37)	Group 3 Magnesium (n = 40)	*p* value^a^
Age (< 50 /≥ 50 years)	27/11	19/18	27/13	0.766
Male/female	26/12	30/7	32/8	0.234
BMI (< 20 /≥ 20–< 30/≥ 30 kg/m)	9/23/6	6/26/5	6/31/3	0.112
**Clinical**				
Recent weight loss (Yes/No)	23/12	30/6	25/12	0.884
Gait disturbance (Yes/No)	32/5	33/4	30/10	0.170
Alcohol intake^b^ (U/week)	210 (126–280)	210 (144–290)	150 (112–280)	0.424
Smoker (Yes/No)	28/9	29/7	27/12	0.510
FAST (< 9/≥ 9)	1/34	2/30	2/35	0.620
GMAWS (at presentation)	4 (2–4)	4 (3–5)	4 (3–4)	0.192
GMAWS (at presentation) (< 4 /≥ 4)	17/20	13/23	19/19	0.718
GMAWS (max)	4 (3–6)	5 (3–6)	4 (2–5)	0.202
GMAWS max (< 4 /≥ 4)	13/25	9/27	17/23	0.431
BDZ total (mg)	140 (38–284)	160 (50–260)	130 (50–240)	0.785
BDZ total (< 120 /≥ 120 mg)	16/18	15/20	19/21	0.954
Time to GMAWS = 0 (h)	10 (6–18)	5.5 (3–8)	6 (3–9)	< 0.001
BDZ to GMAWS = 0 (Diazepam (mg))	50 (38–105)	40 (30–70)	35 (30–70)	0.060
**Medication**				
Thiamine (yes/no)	32/6	30/6	26/13	0.064
Magnesium (yes/no)	1/35	2/32	5/34	0.096
PPi (yes/no)	27/10	19/17	22/17	0.146
H2 Blocker (yes/no)	2/33	2/32	1/36	0.545
**Profile of hospital attendance**				
ED presentation within 1 month (yes/no)	10/27	9/26	7/32	0.350
ED presentations within 12 months	3 (1–9)	3 (1–5)	2 (1–5)	0.779
Total number of admissions	14 (5–42)	14 (8–34)	17 (6–29)	0.982

Continuous data are presented as median and interquartile range (parentheses).

^a^Categorical and continuous data were analysed with Chi-squared and Kruksall-Wallis tests respectively.

^b^Group 1: n = 19; Group 2: n = 16; Group 3: n = 21.

**Table 2:** (a) Pre- and post- treatment laboratory results for randomisation groups (n = 115), (b) Sensitivity analysis: Pre- and post- treatment laboratory results according to randomisation groups for patients receiving long-term treatment with oral thiamine supplementation prior to recruitment (n = 88).

	Group 1 Thiamine (n = 38)	Group 2 Thiamine and Magnesium (n = 37)	Group 3 Magnesium (n = 40)	*p* value^a^
**(a)**				
**Erythrocyte TDP (< 275**/**≥ 275**–**675**/**> 675 ng/gHb)**				
Pre	1/20/17	0/22/14	0/24/12	0.263
	631 (460–929)	644 (553–815)	577 (453–764)	0.302
Post	0/1/30**	0/2/33**	0/24/12	< 0.001
	1056 (895–1213)	1066 (908–1186)	564 (435–789)	< 0.001
**Erythrocyte magnesium (≥**/**< 6.5 mmol/L)**				
Pre	31/6	25/9	30/9	0.478
	7.4 (6.7–7.6)	7.2 (6.5–7.7)	7.3 (6.7–7.9)	0.901
Post	28/4	25/8	28/8	0.331
	7.5 (6.6–7.7)	7.2 (6.5–7.7)	7.3 (6.5–7.9)	0.765
**Serum magnesium (≥**/**< 0.75 mmol/L)**				
Pre	15/23	14/20	17/22	0.715
	0.71 (0.56–0.78)	0.71 (0.62–0.81)	0.70 (0.62–0.81)	0.605
Post	12/21	29/4**	30/4**	< 0.001
	0.67 (0.54–0.79)	0.96 (0.88–1.1)	0.92 (0.85–1.0)	< 0.001
**ETKA basal (U/gHb)**				
Pre	0.66 (0.54–0.76)	0.65 (0.47–0.82)	0.62 (0.50–0.73)	0.442
Post	0.76 (0.56–0.93)	0.60 (0.51–0.84)	0.63 (0.51–0.71)	0.102
**ETKA basal (≥**/**< 0.6 U/gHb)**				
Pre	24/10	17/14	19/15	0.217
Post	24/8	17/14	23/11	0.549
**ETKA TDP activation (U/gHb)**				
Pre	0.60 (0.49–0.68)	0.58 (0.46–0.74)	0.57 (0.46–0.69)	0.819
Post	0.67 (0.49–0.78)	0.57 (0.49–0.71)	0.61 (0.47–0.72)	0.484
**ETKA (<**/**≥ 15% TDP activation)**				
Pre	29/2	23/5	29/4	0.500
Post	29/1	24/4	29/4	0.256
**Glucose (< 7**/**≥ 7 mmol/L)**				
Pre	26/12	24/10	26/14	0.743
	6.1 (5.4–7.6)	6.1 (5.4–7.6)	5.9 (4.9–7.8)	0.782
Post	20/7	24/8	26/8	0.828
	5.8 (4.9–7.0)	5.9 (5.2–7.0)	5.6 (4.9–6.9)	0.510
**LDH (<**/**≥ 240 U/L)**				
Pre	6/16	10/10	11/16	0.375
	302 (233–380)	233 (203–309)	251 (217–328)	0.141
Post	8/13	11/11	13/12	0.359
	264 (202–297)	238 (198–278)	236 (209–295)	0.825
**Lactate (≤ 2.0**/**> 2.0 mmol/L)**				
Pre	14/23	10/26	14/26	0.808
	2.4 (1.5–4.5)	2.9 (1.7–4.9)	2.7 (1.6–4.4)	0.834
Post	19/15	26/9**	22/16*	0.904
	1.5 (1.1–3.3)	1.2 (0.9–2.4)	1.8 (1.1–2.7)	0.384
**H**^**+**^** (< 35**/**≥ 35–< 45**/**≥ 45 nmol/L)**				
Pre	14/13/4	12/17/3	9/25/0	0.004
	36 (32–39)	37 (31–40)	38 (34–40)	0.344
Post	9/12/2	8/21/1	4/26/0	0.004
	36 (32–40)	38 (32–41)	40 (38–42)	0.018
**HCO**_**3**_^**−**^** (< 20 /20–30 **/**> 30 mmol/L)**				
Pre	3/21/9	2/17/13	1/25/9	0.658
	29 (25–31)	29 (25–34)	26 (24–31)	0.259
Post	1/13/9	1/13/16	1/19/10	0.558
	29 (26–33)	31 (27–33)	26 (24–30)	0.029

	Group 1 Thiamine (n = 32)	Group 2 Thiamine and Magnesium (n = 30)	Group 3 Magnesium (n = 26)	*p* value^a^
**(b)**				
**Erythrocyte TDP (< 275 **/**≥ 275**–**675**/**> 675 ng/gHb)**				
Pre	0/15/17	0/16/14	0/13/11	0.575
	693 (480–960)	661 (557–850)	606 (460–826)	0.628
Post	0/0/25***	0/1/28***	0/13/11	< 0.001
	1060 (917–1344)	1099 (935–1262)	616 (476–836)	< 0.001
**Erythrocyte magnesium (≥**/**< 6.5 mmol/L)**				
Pre	25/6	21/8	20/5	0.913
	7.4 (6.5–7.7)	7.3 (6.5–7.7)	7.3 (6.7–8.2)	0.808
Post	23/4	22/7	18/6	0.369
	7.5 (6.9–7.8)	7.3 (6.5–7.7)	7.3 (6.5–8.1)	0.724
**Serum magnesium (≥**/**< 0.75 mmol/L)**				
Pre	13/19	12/16	9/16	0.746
	0.71 (0.56–0.78)	0.72 (0.61–0.82)	0.68 (0.50–0.82)	0.557
Post	11/18	25/3***	19/4***	< 0.001
	0.67 (0.54–0.79)	0.98 (0.88–1.1)	0.91 (0.76–0.99)	< 0.001
**ETKA basal (U/gHb)**				
Pre	0.71 (0.61–0.81)	0.67 (0.47–0.78)	0.63 (0.58–0.76)	0.563
Post	0.76 (0.62–0.93)	0.62 (0.52–0.84)	0.67 (0.61–0.74)	0.088
**ETKA basal (≥**/**< 0.6 U/gHb)**				
Pre	22/6	15/11	16/7	0.441
Post	21/5	15/11	18/5	0.790
**ETKA TDP activation (U/gHb)**				
Pre	0.62 (0.52–0.70)	0.58 (0.46–0.70)	0.64 (0.50–0.71)	0.658
Post	0.72 (0.59–0.78)	0.57 (0.49–0.72)	0.66 (0.56–0.73)	0.183
**ETKA TDP activation (<**/**≥ 15%)**				
Pre	25/1	20/4	20/3	0.290
Post	24/1	21/3	20/3	0.288
**Glucose (< 7**/**≥ 7 mmol/L)**				
Pre	23/9	19/9	16/10	0.409
	6.0 (5.4–7.3)	5.8 (5.4–7.7)	6.0 (5.0–7.9)	0.997
Post	17/5	20/6	17/5	1.0
	5.7 (4.9–6.9)	6.0 (5.3–6.9)	5.8 (4.9–6.9)	0.570
**LDH (<**/**≥ 240 U/L)**				
Pre	4/12	10/8	5/11	0.718
	302 (243–376)	216 (202–308)	255 (217–324)	0.172
Post	6/9	10/9	7/9	0.847
	249 (206–288)	235 (202–268)	262 (189–297)	0.685
**Lactate (≤ 2.0**/**> 2.0 mmol/L)**				
Pre	13/19	10/20	5/21	0.087
	2.5 (1.4–5.2)	2.6 (1.3–5.0)	3.5 (2.2–5.6)	0.358
Post	16/12	24/5***	10/15	0.243
	1.4 (0.9–2.5)	1.4 (0.9–2.0)	2.3 (1.7–3.4)	0.004
**H**^**+**^** (< 35 **/**≥ 35– < 45 **/**≥ 45 nmol/L)**				
Pre	12/10/3	11/13/2	5/19/0	0.004
	36 (32–39)	37 (31–41)	39 (36–41)	0.153
Post	6/11/1	6/17/1	1/20/0	0.015
	37 (34–40)	38 (33–41)	40 (39–41)	0.014
**HCO**_**3**_^**-**^** (< 20 /20–30 **/**> 30 mmol/L)**				
Pre	3/16/8	1/16/9	1/18/6	0.474
	29 (24–31)	28 (25–34)	25 (24–31)	0.341
Post	1/9/8	1/10/13	0/14/7	0.350
	29 (26–35)	31 (26–33)	26 (24–31)	0.211

^a^Categorical and continuous data were analysed with Chi-squared and Kruksall-Wallis tests respectively.

*,**Changes between pre- and post- treatment measurements with significance *p* < 0.01 and *p* < 0.001 respectively as calculated by Wilcoxon Signed Ranks test.

***Changes between pre- and post- treatment measurements with significance *p* < 0.001 as calculated by Wilcoxon Signed Ranks test.

Of the laboratory analysis, the majority of patients had bilirubin (58%), alkaline phosphatase (68%), AST/ALT ratio < 2 (65%), albumin (77%), CRP (57%), urea (68%), sodium (71%), potassium (82%), MCV (51%) and platelets (70%) within the laboratory reference interval.

**Figure 2:**
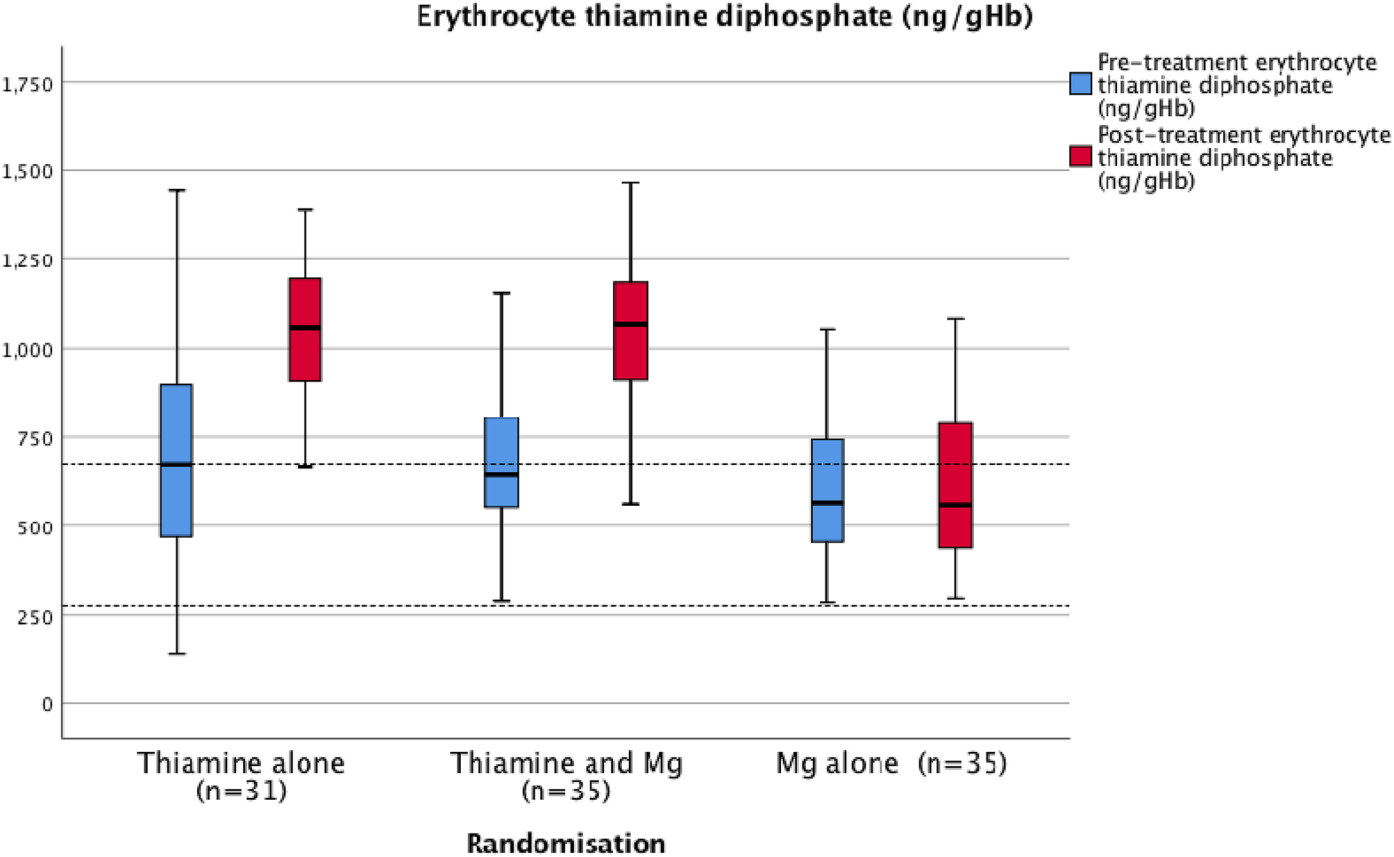
Pre- and post-treatment erythrocyte thiamine diphosphate concentrations.

Prior to recruitment to the trial, the majority of patients had been prescribed long-term oral thiamine supplementation (78%) in the outpatient setting. It was of interest that only one of the patients recruited to the trial had erythrocyte thiamine concentration below the reference range (< 275 ng/gHb) (< 1%) prior to randomisation and treatment. Prior to randomisation and treatment, the majority of patients (59%) had erythrocyte thiamine diphosphate concentrations within the normal reference range (≥ 275–675 ng/gHb) and 40% of patients had erythrocyte thiamine diphosphate concentrations above the reference range (> 675 ng/gHb) (see Fig. 2). One third of patients (33%) who had erythrocyte thiamine diphosphate concentrations above the reference range (> 675 ng/gHb) at the time of trial recruitment had been admitted to hospital and treated with parenteral thiamine (Pabrinex®) within one month prior to the trial related admission. It was of interest that the magnesium alone treatment group (Group 3) (Table 1, group 3) had a trend towards lesser prior thiamine supplementation (*p* = 0.064) (Table 2a) and therefore post hoc analysis of laboratory results were carried out (see Table 2b). The significance of the results were similar.

**Figure 3:**
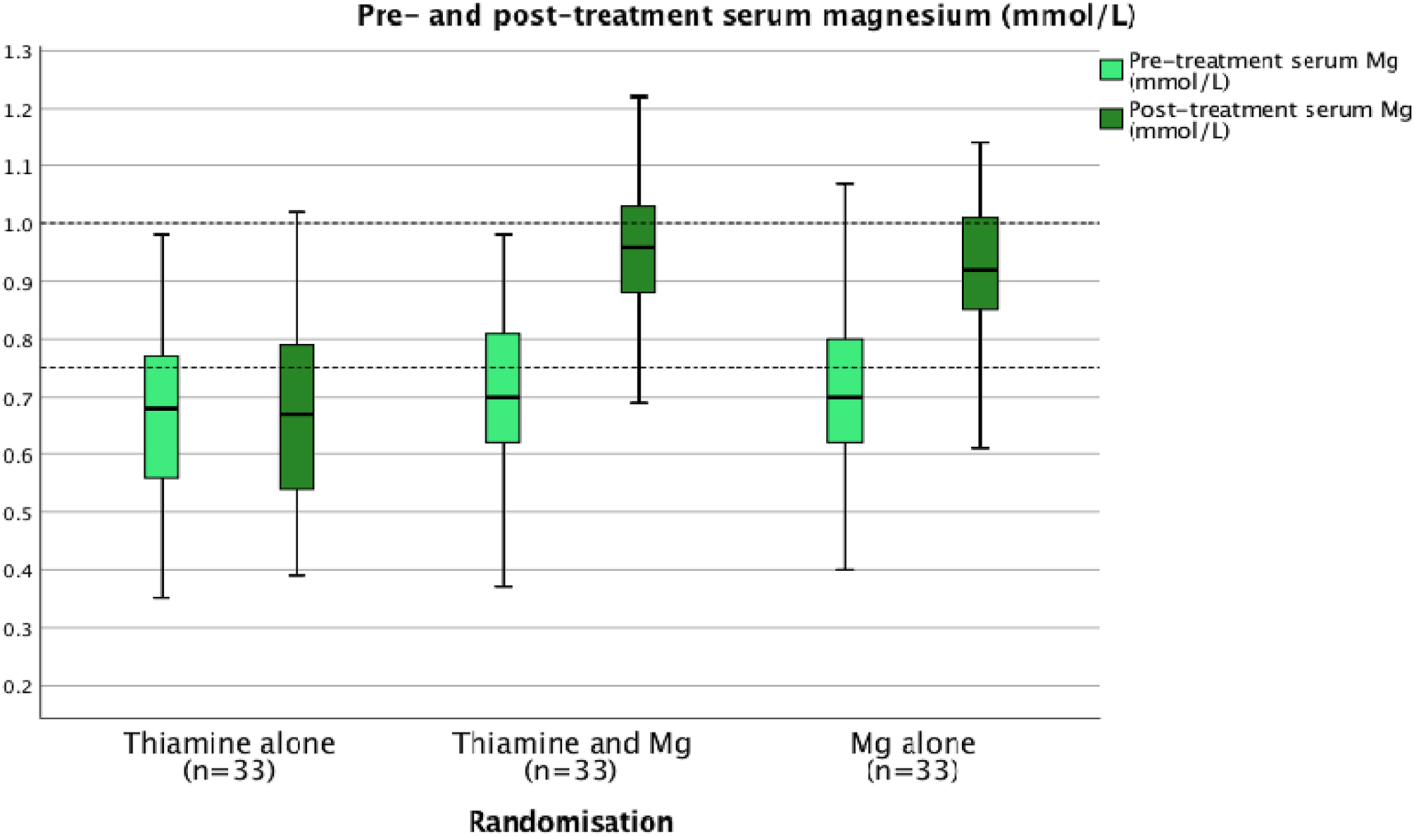
Pre- and post-treatment serum magnesium concentrations according to randomisation.

In contrast to the baseline thiamine status of the patients recruited to the randomised controlled trial, the majority of patients (59%) had circulating magnesium concentrations that could be considered to be low (< 0.75 mmol/L) (Fig. 3) and had not been prescribed magnesium (93%) prior to randomisation and treatment. The median baseline serum magnesium concentration (0.70 mmol/L) was observed to be below the lower limit of the reference range (< 0.75 mmol/L) across all three randomisation groups (n = 111) (Fig. 3).

Following treatment, patients randomised to group 1 (thiamine alone treatment group) and group 2 (combined thiamine and magnesium treatment group) were observed to increase erythrocyte thiamine diphosphate concentrations (group 1: median absolute delta = 393 ng/gHb; IQR 256–503 ng/gHb; n = 31 and group 2: median absolute delta = 324 ng/gHb; IQR 247–528 ng/gHb; n = 35) (Fig. 2, Table 2a). Following treatment, 86% of patients who received thiamine (groups 1 and 2) were observed to have erythrocyte thiamine diphosphate concentrations above the upper limit of the reference range (> 675 ng/gHb).

Following treatment the median serum magnesium concentration was 0.67 mmol/L, 0.96 mmol/L, and 0.92 mmol/L for groups 1, 2 and 3 respectively (*p* < 0.001) (see Table 2a). Patients randomised to both group 2 (combined thiamine and magnesium treatment group) and group 3 (magnesium alone treatment group) were observed to increase serum magnesium concentrations (group 2: median absolute delta = 0.25 mmol/L; IQR 0.21–0.29 mmol/L; n = 33 and group 3: median absolute delta = 0.26 mmol/L; IQR 0.18–0.33 mmol/L; n = 33) (*p* < 0.001) (Table 2a, Fig. 3). Furthermore, it was of note that patients in these treatment groups (2 and 3), who received 2 g magnesium sulphate over 30 min, did not reach serum magnesium concentrations in the toxic range (> 2 mmol/L) (Fig. 3).

**Figure 4:**
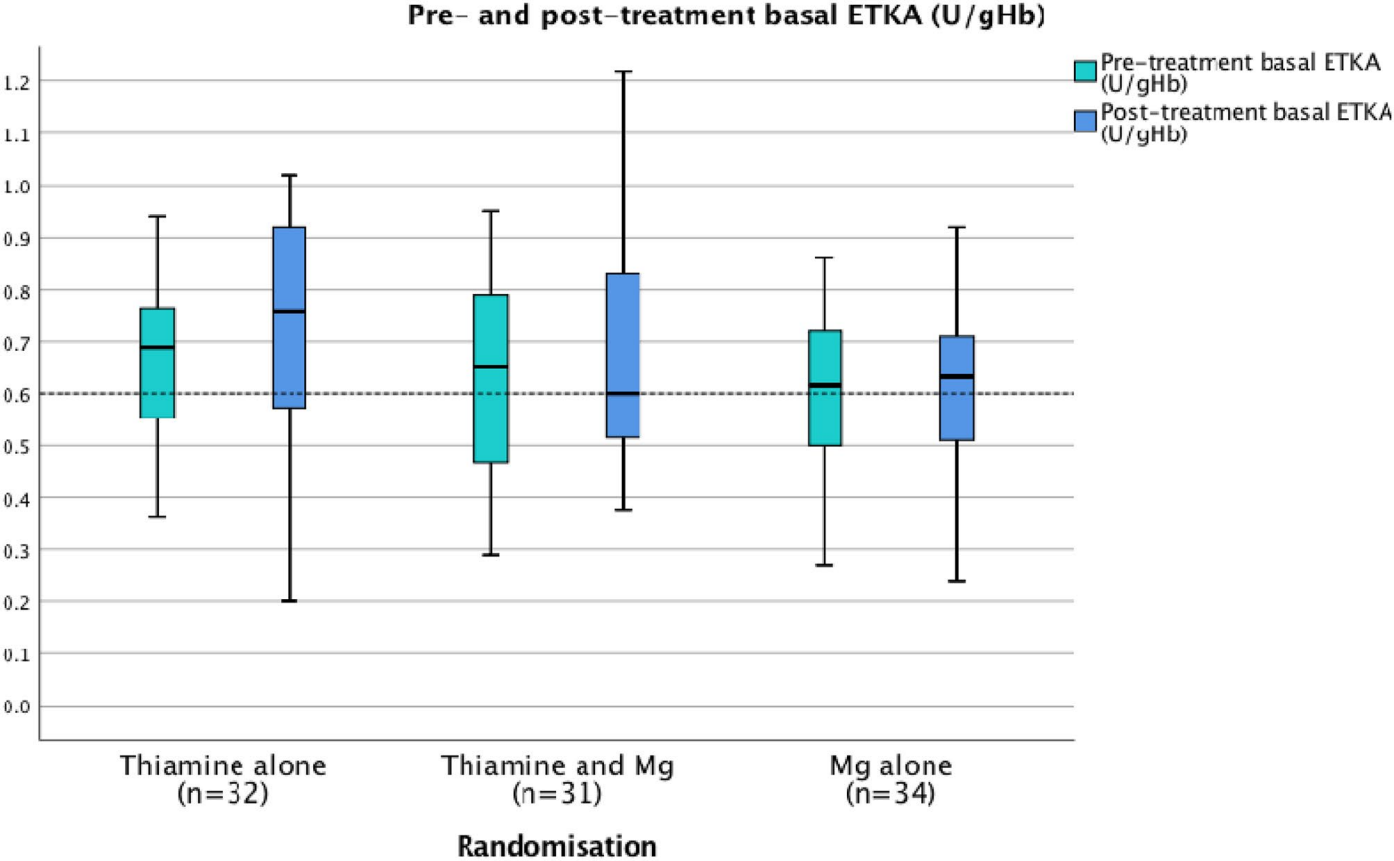
Pre- and post-treatment basal ETKA (n = 93).

The primary outcome measure, erythrocyte transketolase activity (ETKA), was not observed to change significantly in group 1 (thiamine alone group), group 2 (thiamine and magnesium group) or group 3 (magnesium alone group) (Fig. 4; Tables 2a, 3).

**Table 3:** Inter-group differences between pre- and post-treatment ETKA according to randomisation.

(I) Randomisation	(J) Randomisation	Mean difference (I–J)	SE	Sig	95% confidence interval	
					Lower bound	Upper bound
1	3	0.11541	0.04955	0.041	0.0040	0.2268
2	3	0.04964	0.04996	0.511	− 0.0627	0.1619

One-way ANOVA Dunnett t-test for inter-group differences, treating group 3 as control, and comparing groups 1 and 2 against it.

**Figure 5:**
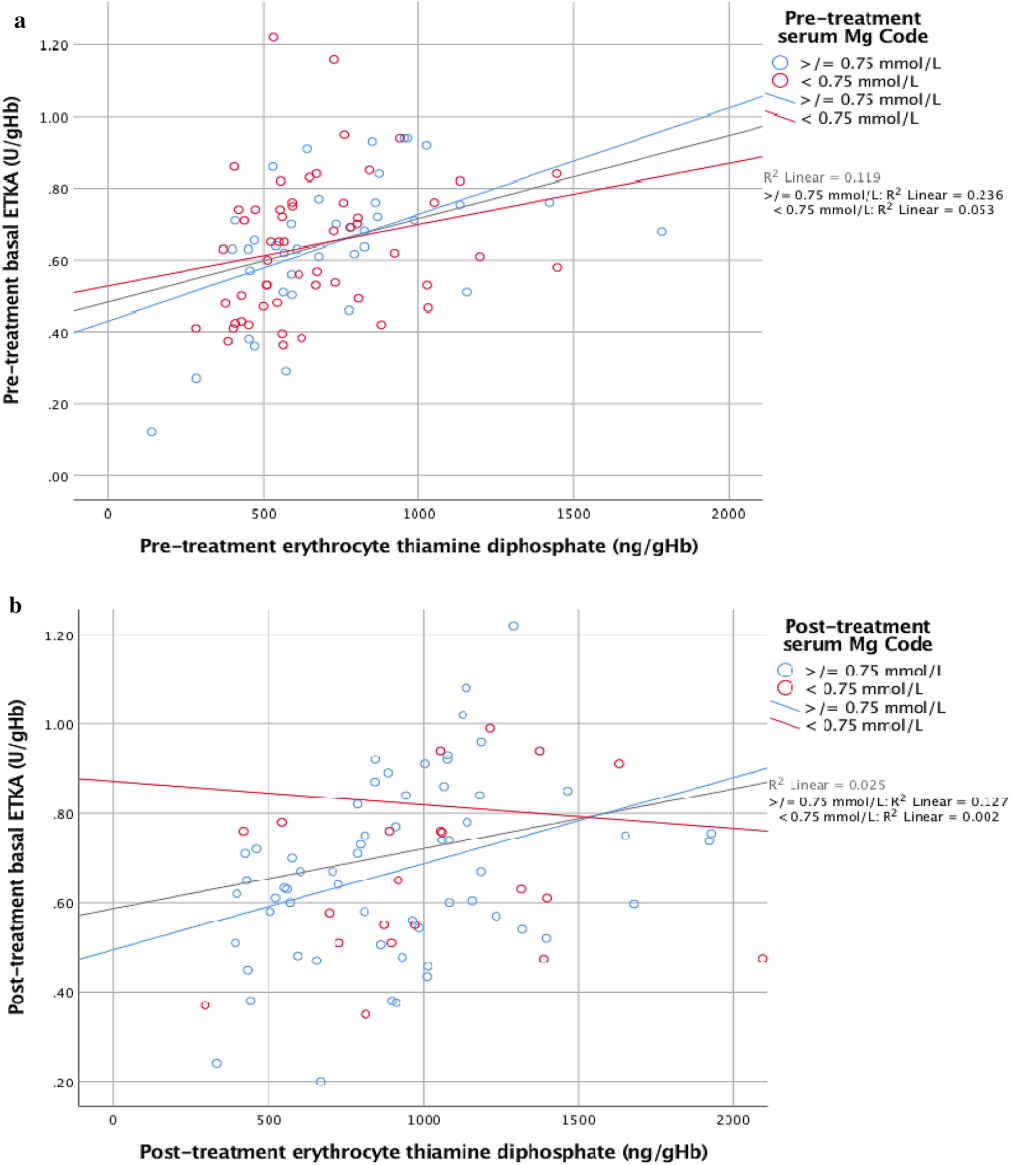
(**a**) Pre-treatment basal ETKA versus pre-treatment erythrocyte TDP with pre-treatment magnesium status highlighted (n = 97), (**b**) Post-treatment basal ETKA versus post treatment erythrocyte TDP with post-treatment magnesium status highlighted (n = 96).

**Figure 6:**
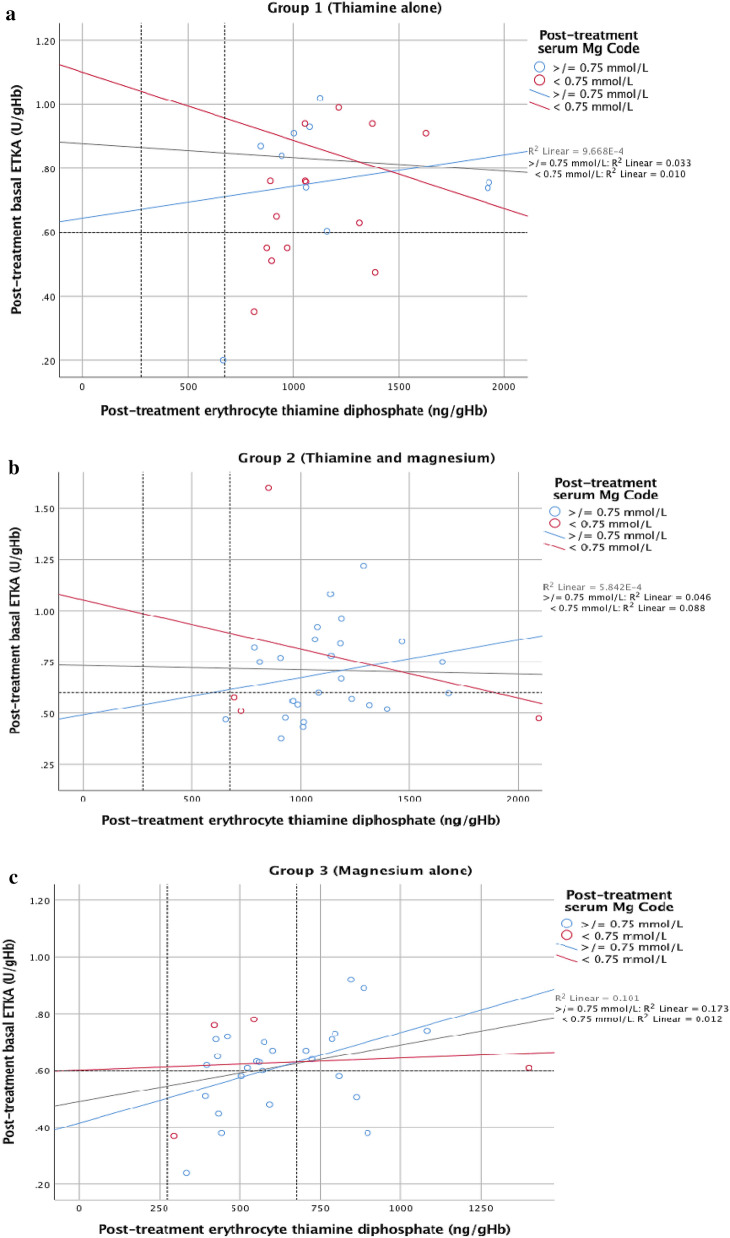
(**a**) Group 1 post-treatment basal ETKA versus erythrocyte TDP (n = 33), (**b**) Group 2 post-treatment basal ETKA versus erythrocyte TDP (n = 31), (**c**) Group 3 post-treatment basal ETKA versus erythrocyte TDP (n = 32).

Patients who had low baseline serum magnesium concentrations (< 0.75 mmol/L) demonstrated less correlation between pre-treatment basal ETKA and erythrocyte thiamine diphosphate concentrations than patients who had serum magnesium concentrations in the normal range (≥ 0.75 mmol/L) (pre-treatment R^2^ = 0.053 and R^2^ = 0.236 respectively) (Fig. 5a). Similarly, those patients who had low post-treatment serum magnesium concentrations (< 0.75 mmol/L) demonstrated less correlation between post-treatment basal ETKA and erythrocyte thiamine diphosphate concentrations than patients who had post-treatment serum magnesium concentrations in the normal range (≥ 0.75 mmol/L) (R^2^ = 0.002 and R^2^ = 0.127 respectively) (Figs. 5b, 6a–c).

Of the laboratory analysis of substrates and products of intermediary metabolism, the median plasma glucose concentration for majority of patients (68%) was within the normal range (median = 6.0 mmol/L; IQR 5.3–7.5 mmol/L; n = 112) prior to randomisation and treatment (Table 2a). Two patients had plasma glucose concentrations below the reference range (< 4.0 mmol/L) prior to randomisation and treatment (3.8 and 3.9 mmol/L respectively). Following treatment, the majority of patients (73%) had plasma glucose concentrations that were within the normal range (median = 5.8 mmol/L; IQR 5.0–7.0 mmol/L; n = 105) and one patient had a plasma glucose concentration below the reference range (3.8 mmol/L). The median absolute change between pre- and post- treatment plasma glucose concentrations was not significant (median plasma glucose absolute delta: group 1 = − 0.5 mmol/L (IQR = − 1.4–0.1 mmol/L), (n = 27); group 2 = − 0.3 mmol/L (IQR = − 1.5–0.3 mmol/L), (n = 32); group 3 = − 0.5 mmol/L (IQR = − 1.9–0.4 mmol/L), (n = 34)) (*p* = 0.843).

It was of interest that pre-treatment plasma glucose ≥ 7.0 mmol/L was significantly associated with pre-treatment plasma lactate concentration > 2.0 mmol/L (n = 105) (*p* < 0.001), GMAWS max ≥ 4 (n = 110) (*p* = 0.009) and length of hospital stay > 5 days (n = 104) (*p* = 0.020).

**Figure 7:**
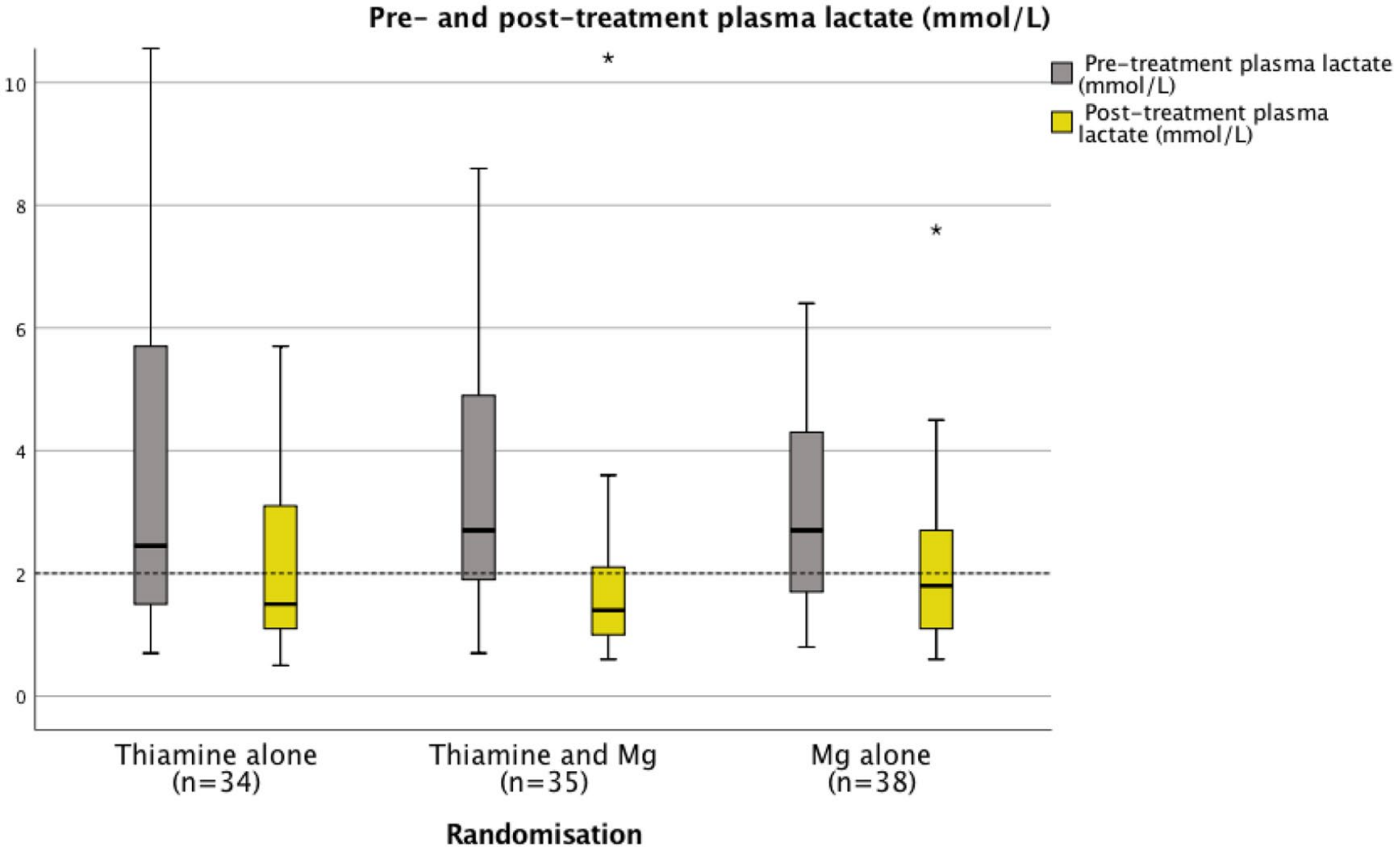
Pre- and post-treatment plasma lactate (all patients).

Prior to randomisation and treatment, the majority of patients (67%) were found to have plasma lactate concentrations > 2 mmol/L, and the median concentration of plasma lactate was > 2 mmol/L across all three randomization groups. Prior to treatment, 64%, 72% and 67% of patients in groups 1, 2 and 3 respectively had plasma lactate concentrations > 2 mmol/L. Following treatment, 44%, 26% and 42% of patients in groups 1, 2 and 3 respectively had plasma lactate concentrations > 2.0 mmol/L (see Fig. 7; Table 4). When patients who had experienced a seizure in the 12-h period prior to trial enrolment (n = 29) were excluded from the analysis, the majority (63%) of the remaining patients (n = 74) had plasma lactate concentrations > 2.0 mmol/L prior to randomisation and treatment. The median plasma lactate concentration for this sub-set of patients (i.e. non-seizure related alcohol withdrawal syndrome presentations) was 2.4 mmol/L (IQR 1.6–4.5 mmol/L). The median plasma lactate concentration for patients recruited to the trial with alcohol withdrawal syndrome who had experienced a seizure within 12 h of presentation was 2.5 mmol/L (IQR 1.7–4.6 mmol/L) (n = 29).

**Table 4:** Pre- and post-treatment plasma lactate (all patients).

Lactate	Group 1		Group 2		Group 3	
	Thiamine alone		Thiamine and magnesium		Magnesium alone	
	Pre treatment	Post treatment	Pre treatment	Post treatment	Pre treatment	Post treatment
< 2 mmol/L	13	19	10	26	13	22
> 2 mmol/L	23	15	26	9	26	16
% Normalisation	20		46		25	
McNemar test	0.070		< 0.001		0.004	

**Figure 8:**
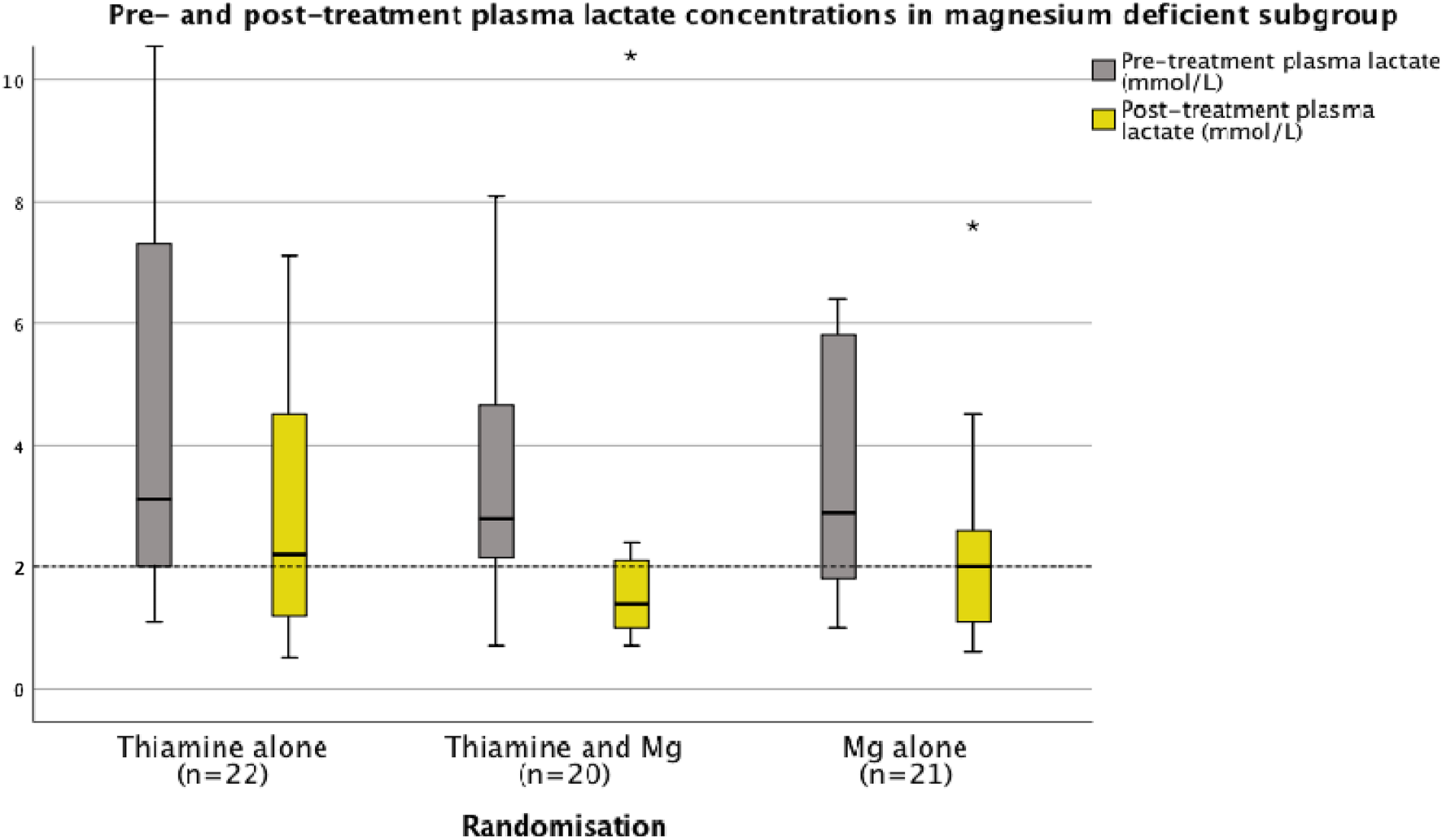
Pre- and post-treatment plasma lactate in the context of low circulating serum magnesium.

At 2 h following treatment median plasma lactate concentrations had normalized (≤ 2.0 mmol/L) across all three randomisation groups (*p* < 0.001 for all groups) (see Table 2a, Fig. 7). However, when randomisation groups were stratified according to pre-treatment magnesium status (</≥ 0.75 mmol/L), patients in group 1 (the thiamine alone group) who had low serum magnesium concentrations (< 0.75 mmol/L) (n = 22) prior to treatment, did not demonstrate significant reduction in plasma lactate concentrations and indeed median plasma lactate concentrations remained above the upper limit of the normal reference interval (> 2.0 mmol/L) in these patients following treatment with thiamine alone (see Fig. 8, Table 5).

**Table 5:** Pre- and post-treatment plasma lactate in the context of low circulating serum magnesium.

Lactate	Group 1		Group 2		Group 3	
	Thiamine alone		Thiamine and magnesium		Magnesium alone	
	Pre treatment	Post treatment	Pre treatment	Post treatment	Pre treatment	Post treatment
< 2 mmol/L	6	9	5	15	6	11
> 2 mmol/L	17	13	15	5	15	10
% Normalisation	15		50		24	
McNemar test	0.375		0.002		0.063	

**Figure 9:**
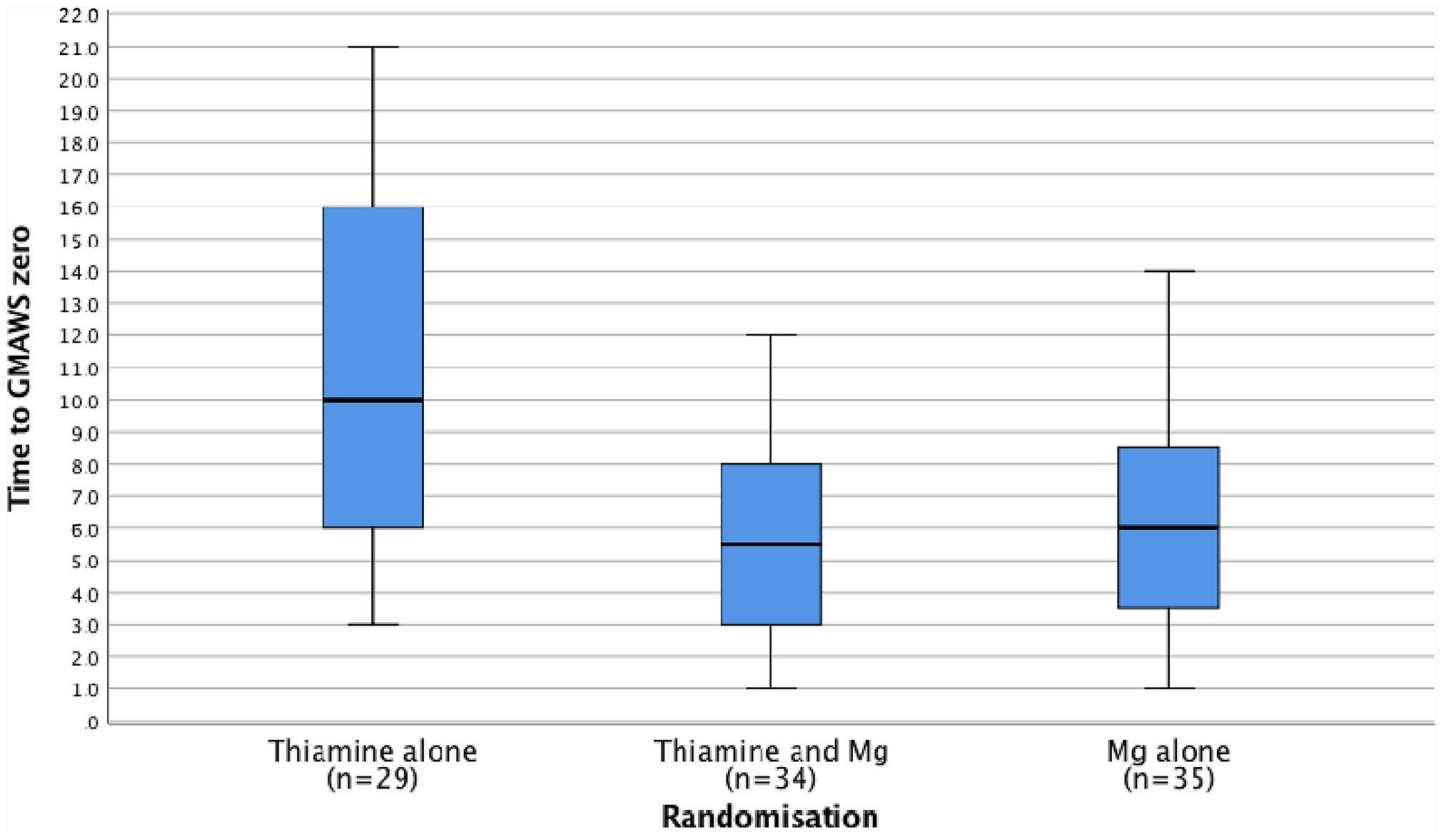
Time (h) to GMAWS = 0 comparison between groups.

Prior to randomisation and treatment, the majority of patients (54%) (n = 61) were found to have GMAWS ≥ 4 (indicating severe alcohol withdrawal) at time of recruitment to the trial (Table 1). The median initial GMAWS scores were 4, 4 and 3.5 for groups 1 (n = 38), 2 (n = 37) and 3 (n = 40) respectively (*p* = 0.192). On secondary analysis, the median time to achieve a GMAWS score = 0 for groups 1, 2 and 3 was 10, 5.5 and 6 h respectively (*p* < 0.001) (Fig. 9, Table 6). When patients were stratified according to pre-treatment magnesium status (</≥ 0.75 mmol/L), the median time for patients with pre-treatment serum magnesium concentrations ≥ 0.75 mmol/L to achieve a GMAWS score = 0 in groups 1 (n = 10), 2 (n = 13) and 3 (n = 13) was 9.5, 4 and 5.5 h respectively (*p* = 0.016). The median time for patients with pre-treatment serum magnesium concentrations < 0.75 mmol/L to achieve a GMAWS score = 0 in groups 1 (n = 19), 2 (n = 19) and 3 (n = 19) was 10, 6 and 6 h respectively (*p* = 0.013).

**Table 6:** Comparison between groups of Time required to achieve first GMAWS = 0 score (one-way ANOVA Tukey test).

(I) Randomisation	(J) Randomisation	Mean difference (I–J)	SE	Sig	95% confidence interval	
					Lower bound	Upper bound
1	2	6.4686	1.4477	0.000	3.022	9.916
	3	6.2719	1.4381	0.000	2.848	9.696
2	1	− 6.4686	1.4477	0.000	− 9.916	− 3.022
	3	− 0.1966	1.3791	0.989	− 3.480	3.087
3	1	− 6.2719	1.4381	0.000	− 9.696	− 2.848
	2	0.1966	1.3791	0.989	− 3.087	3.480

**Figure 10:**
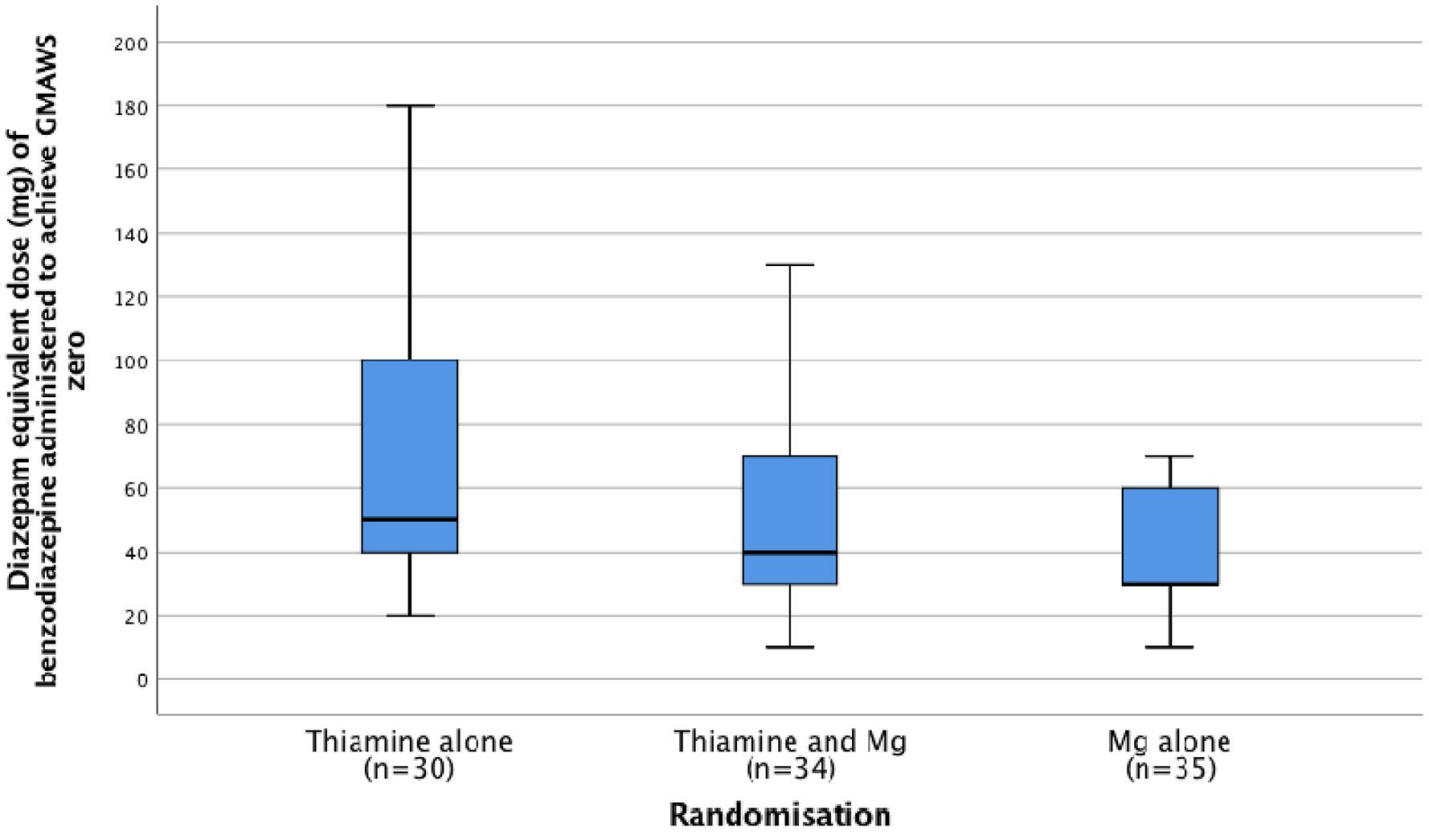
Benzodiazepine (Diazepam equivalent dose) to GMAWS = 0 comparison between groups.

The median diazepam equivalent dose (mg) of benzodiazepine required to achieve the first GMAWS score = 0 for patients in groups 1, 2 and 3 was 50, 40 and 30 mg respectively (*p* = 0.060) (see Fig. 10, Table 7). When patients were stratified according to pre-treatment magnesium status (</≥ 0.75 mmol/L), the median diazepam equivalent dose of benzodiazepine required for patients with pre-treatment serum magnesium concentrations ≥ 0.75 mmol/L to achieve a GMAWS score = 0 in groups 1 (n = 11), 2 (n = 13) and 3 (n = 16) was 50, 30 and 30 mg respectively (*p* = 0.308). The median diazepam equivalent dose of benzodiazepine required for patients with pre-treatment serum magnesium concentrations < 0.75 mmol/L to achieve a GMAWS score = 0 in groups 1 (n = 19), 2 (n = 19) and 3 (n = 18) was 60, 50 and 40 mg respectively (*p* = 0.130).

**Table 7:** Comparison between groups of benzodiazepine (Diazepam equivalent dose) required to achieve first GMAWS = 0 score (one-way ANOVA Tukey test).

(I) Randomisation	(J) Randomisation	Mean difference (I–J)	SE	Sig	95% confidence interval	
					Lower bound	Upper bound
1	2	22.373	11.520	0.133	− 5.05	49.80
	3	21.524	11.442	0.150	− 5.72	48.76
2	1	− 22.373	11.520	0.133	− 49.80	5.05
	3	− 0.849	11.074	0.997	− 27.21	25.51
3	1	− 21.524	11.442	0.150	− 48.76	5.72
	2	0.849	11.074	0.997	− 25.51	27.21

## Discussion

To our knowledge, this is the first trial to compare the efficacy of thiamine and/or magnesium treatment in patients with alcohol withdrawal syndrome. The present trial has demonstrated significant differences between pre- and post-treatment basal ETKA in patients who received thiamine alone (groups 1), however it has not replicated the significant enhancement of ETKA that was measured in the pilot trial^17^.

In the present trial, group 1 (thiamine alone) and group 2 (thiamine and magnesium group) were observed to demonstrate increases in erythrocyte thiamine diphosphate concentrations. It was of interest that only one of the patients was thiamine deficient (< 1%) prior to randomisation and treatment. Following treatment, the majority of patients in group 1 (97%) and group 2 (94%) had erythrocyte thiamine diphosphate concentrations above the reference range (> 675 ng/gHb). In group 3, 30% of patients had pre-treatment erythrocyte thiamine diphosphate concentrations above the reference range and this remained unchanged following randomisation and treatment.

In contrast to thiamine status, the majority of patients (59%) were observed to be magnesium deficient (serum magnesium concentration < 0.75 mmol/L) and the median serum magnesium concentration was observed to be below the lower limit of the reference interval across all three randomisation groups. Patient groups 2 and 3 who received magnesium alone or combined treatment with thiamine and magnesium increased their serum magnesium concentrations and it was of note that neither of these groups reached serum magnesium concentrations in the toxic range.

One potential explanation, for the difference between the pilot study and the present trial in ETKA results, is that some of the patients recruited to the pilot study may have been thiamine and/or magnesium deficient, whereas 99% of patients recruited to the present trial were thiamine replete.

Nonetheless, it was of interest that ETKA was activated > 15% in some of the patients in the present trial who had normal direct measurements of pre-treatment erythrocyte thiamine diphosphate concentrations (Table 2a). This observation was previously reported among chronic AUD patients and attributed to structural enzymatic changes^28^, however no consideration had been given to the potential role of co-existent magnesium deficiency when this assertion was made.

The present randomised trial gives an insight into the activity of ETKA in the presence of low and normal magnesium status in both in vitro and in vivo settings. When pre-treatment basal ETKA results were analysed in the context of magnesium status, it was apparent that the activity of ETKA following stimulation with TDP was significantly less among the magnesium depleted groups as compared to the magnesium replete groups (Fig. 5a).

ETKA fell out of favour as a measure of thiamine status in the late 1990’s as it had been reported to be difficult to standardize, resulting in significant inter-laboratory variability. This difficulty in standardization and significant inter-laboratory variability may have been attributable to variation in baseline magnesium status in individual samples. Although routine ETKA measurement has been superseded by the advent of high performance liquid chromatography (HPLC) that enabled reliable and direct measurement of TDP in erythrocyte samples^29,30^, such measurements are currently batch analysed and therefore not available to the clinician when deciding whether or not to administer thiamine.

Furthermore, it is well established in the biochemistry laboratory setting that the activity of transketolase is dependent on the presence both TDP and magnesium^17,28,31^ and some have suggested that it was the activity of thiamine, which was relevant, rather than the finite mass available^32^. On this basis, some authorities continue to recommend the measurement of baseline and TDP enhanced ETKA as the gold standard for the accurate quantification of intracellular thiamine status^33^. However, to date this laboratory based knowledge has not translated into evidenced based clinical practice.

In the present trial, when ETKA results are analysed in the context of magnesium status, it is apparent that basal ETKA relative to erythrocyte thiamine diphosphate concentrations is significantly less among the magnesium depleted groups as compared to the magnesium replete groups (see Figs. 5a, 6c). Given that the majority of patients with AWS in this cohort have low serum magnesium concentrations and the significant association of low serum magnesium concentrations with increased mortality^4^, the results of the present trial may offer an insight into one potential mechanism by which low circulating magnesium status may result in suboptimal thiamine dependent enzyme activity and deranged intermediary metabolism.

Prior to treatment, the majority of patients (67%) had plasma lactate concentrations > 2.0 mmol/L and the median plasma lactate concentration was > 2.0 mmol/L across all three randomization groups. This observation persisted when patients who had experienced a seizure in the 12-h period prior to the trial related presentation were excluded from the analysis. Furthermore, it was of interest that there was a significant normalization of plasma lactate concentrations in those patients who were initially magnesium deficient in the thiamine and magnesium (Group 2) and the magnesium alone (Group 3) groups. In contrast, plasma lactate concentrations did not normalise among magnesium deficient patients in the thiamine alone group (Group 1) and median plasma lactate concentrations were observed to remain above the upper limit of the reference interval (> 2.0 mmol/L) among magnesium deficient patients in this group (Group 1) (see Fig. 8). The consistent normalization of plasma lactate concentrations observed in the combined thiamine and magnesium treatment group (Group 2) may have the potential to influence evidence based clinical protocols in the care of patients experiencing alcohol withdrawal syndrome as a significant association was found between elevated pre-treatment lactate concentrations and severe alcohol withdrawal scores (GMAWS ≥ 4).

This trial presents the largest cohort of AWS patients to have non-seizure related elevated plasma lactate concentrations recorded to date and as such elevated plasma lactate concentrations may have further use as a predictor of likelihood of development of severe AWS. Furthermore, given that sustained elevation of plasma lactate concentrations are widely recognized by clinicians as an adverse prognostic indicator in both general and critical care clinical settings^34^, the consistent normalization of plasma lactate concentrations demonstrated among patients in the present trial who received combined thiamine and magnesium treatment is worthy of investigation in other clinical conditions (e.g. Systemic Inflammatory Response Syndrome)*.*

Inhibitory gamma-aminobutyric acid (GABA) and excitatory N-methyl-D-aspartate (NMDA) neurotransmitter receptors are implicated in the pathogenesis of alcohol withdrawal syndrome and seizures^35,36^. The activity of these receptors is interdependently balanced. NMDA activity is mediated by glutamate^37,38^. Alcohol directly enhances GABA receptor complex activity and chronic alcohol consumption results in down-regulation of GABA receptors^36^. Chronic excess alcohol consumption also generates increased glutamate production and mediates up-regulation of NMDA receptor activity^35,39^. Hence, when a ‘relative deficiency’ of alcohol occurs in a chronic alcohol user, NMDA activity predominates causing symptoms of alcohol withdrawal to manifest and, if left untreated, seizures to occur^35,36,40^. The data presented in the present trial would suggest that those patients who received magnesium (Groups 2 and 3) experienced faster initial resolution of AWS than those in Group 1 who received thiamine alone. Hence, the combined administration of thiamine and magnesium to patients with AWS related elevated plasma lactate in the context of low circulating magnesium concentrations may have therapeutic potential in ameliorating the duration/severity of AWS experienced by patients. However, despite the significant reduction in time to initial resolution of AWS, no significant benzodiazepine sparing effect was shown in the data. This raises the possibility that other micronutrients that influence the stability of the NMDA receptor may also be deficient in this patient group e.g. zinc.

It is also of interest that essential neurotransmitters (glutamate, GABA, acetylcholine) are produced by Krebs cycle^41^. Thus, reduced GABA production may be attributable to both direct and indirect mechanisms of alcohol related compromise of Krebs cycle. However, to the best of our knowledge, there are no studies reporting an association between parenteral thiamine administration and treatment of AWS symptoms. While it is possible that the delayed administration of thiamine to patients in treatment Group 3 may have delayed enhanced GABA production by optimisation of thiamine dependent enzyme function, this is not reflected in the data. Moreover, NMDA receptor channels are reported to exhibit blockade by physiological concentrations of magnesium and a high affinity voltage dependent magnesium-binding site has been shown to regulate the activity of this receptor channel^42^. NMDA receptors therefore require magnesium to maintain stability and integrity^43,44^, and low magnesium concentrations are known to up-regulate NMDA receptor activity, lowering seizure thresholds^44,45^. Hence, the reduction in time to achieve initial GMAWS = 0 in the groups that received parenteral MgSO4 (Groups 2 and 3) compared to the group that received thiamine alone (Group 1) may be attributable to the direct stabalizing effect of Mg^2+^ on the NMDA receptor channel.

Overall, this therapeutic approach warrants further exploration as it remains to be conclusively determined if co-administration of thiamine and magnesium will result in faster resolution of elevated plasma lactate concentrations, reduced duration and/or severity of AWS symptoms and better patient outcomes for patients with alcohol use disorder who have deficiency of these cofactors and experience AWS.

### Limitations

Pabrinex® is a multivitamin preparation, containing thiamine, riboflavin, pyridoxine, ascorbic acid, nicotinamide, and glucose. Given the role of riboflavin as a co-factor for pyruvate dehydrogenase, a potential limitation of the present trial is that pre- and post-treatment riboflavin concentrations have not been measured as this is a potential confounding factor for the interpretation of plasma lactate concentrations.

## Conclusion

Only Group 1 (thiamine alone treatment group) demonstrated significant enhancement of ETKA (the primary endpoint), however post hoc analysis demonstrates significant enhancement of ETKA among patients with serum magnesium concentrations > 0.75 mmol/L relative to patients with serum magnesium < 0.75 mmol/L. The findings of low serum magnesium concentrations (< 0.75 mmol/L) and elevated plasma lactate concentrations have implications for patients with AWS. As such, these investigations should form part of the routine investigations undertaken for patients presenting to the Emergency Department with alcohol use disorder. Overall, co-administration of thiamine and magnesium resulted in more consistent normalization of plasma lactate concentrations and faster initial resolution of AWS symptoms. It remains to be determined if this treatment strategy may translate into meaningful clinical outcomes for patients experiencing alcohol withdrawal syndrome.

## Supplementary Information

Supplementary Information.
