# Supplementary material for: Randomised trial of intravenous thiamine and/or magnesium sulphate administration on erythrocyte transketolase activity, lactate concentrations and alcohol withdrawal scores
Source: Sci Rep. 2022 Apr 28;12:6941. doi: 10.1038/s41598-022-10970-x (PMC9051209; doi:10.1038/s41598-022-10970-x)
Supplement: Supplementary file 1 — Supplementary Information. [file 41598_2022_10970_MOESM1_ESM.docx]

**
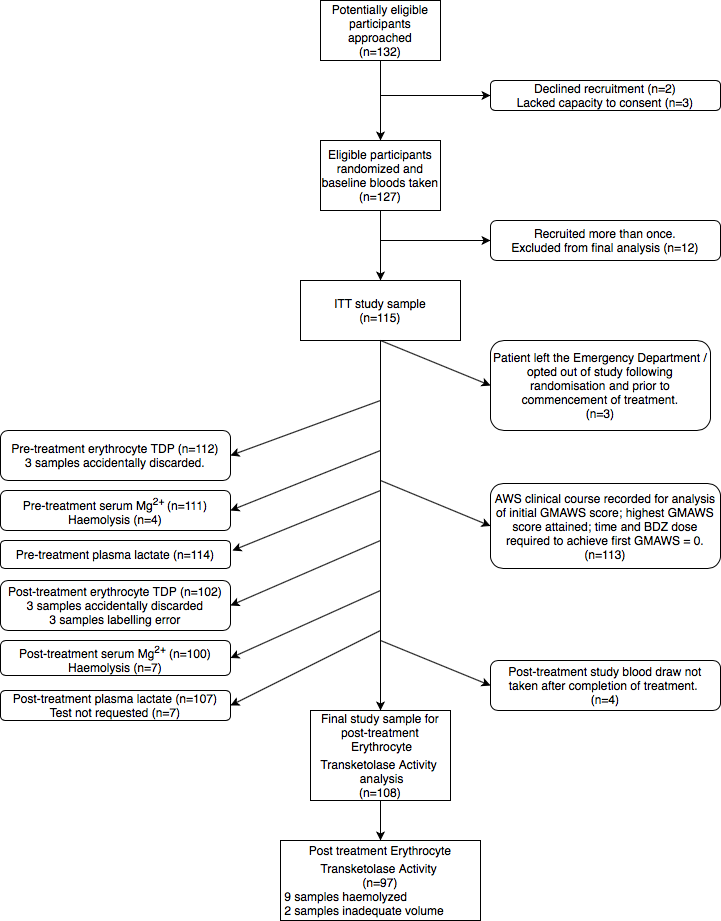
**

**Appendix A.** Consort Diagram detailing patient recruitment and participation in randomised controlled trial
